# Application of an artificial-intelligence–based transesophageal echocardiography simulation system in residency training

**DOI:** 10.3389/fsurg.2026.1762740

**Published:** 2026-05-25

**Authors:** Meng Zhang, Si-Yuan Song, Huixian Zhou, Guangmin Xu, Xianxiang Tong, Dan Fan, Qian Lei

**Affiliations:** 1 Department of Anesthesiology, Sichuan Provincial People's Hospital, University of Electronic Science and Technology of China, Chengdu, Sichuan, China; 2 Department of Anesthesiology, Montefiore Medical Center, Albert Einstein College of Medicine, Bronx, NY, United States; 3 Department of Neuroscience, Baylor College of Medicine, Houston, TX, United States; 4 Department of Internal Medicine, Montefiore Medical Center Wakefield Campus, Bronx, NY, United States

**Keywords:** anesthesiology, artificial intelligence, residency education, simulation training, transesophageal echocardiography

## Abstract

**Background:**

Transesophageal echocardiography (TEE) is a key perioperative monitoring modality in anesthesiology, but traditional training is constrained by limited case exposure, patient safety concerns, and heterogeneous learning curves. Artificial-intelligence (AI)–based TEE simulators that integrate 3-dimensional (3D) anatomy visualization, interactive image interpretation, and virtual probe manipulation may improve learning efficiency and confidence in residents.

**Methods:**

In this single-center randomized controlled study, sixty anesthesiology residents in standardized training at Sichuan Academy of Medical Sciences & Sichuan Provincial People's Hospital (December 2024–December 2025) were randomly allocated to an AI group (*n* = 30) or a control group (*n* = 30). Both groups received the same instructor-led TEE curriculum. The control group underwent traditional teaching (lectures, static images, and video demonstrations), whereas the AI group additionally trained on an AI-based TEE simulation system that provided 3D anatomy visualization, interactive image-reading exercises, and virtual probe operation with real-time feedback. Outcomes included a standardized image-interpretation test (0–100), Objective Structured Assessment of Technical Skills for TEE (OSATS-TEE; 0–150), key-view acquisition rate, procedure time, self-efficacy score, and course satisfaction (all 0–100).

**Results:**

Baseline demographic characteristics did not differ between groups (all *p* > 0.05). After the course, the AI group achieved higher image-interpretation test scores than the control group (84.3 ± 6.9 vs. 78.1 ± 7.8, *p* = 0.002) and higher scores on the training-context-specific OSATS-TEE assessment (121.5 ± 9.2 vs. 112.4 ± 10.1, *p* < 0.001). The key-view acquisition rate was significantly higher in the AI group (88.2% ± 8.1% vs. 76.5% ± 9.7%, *p* < 0.001), while mean operation time was shorter (27.1 ± 3.9 min vs. 32.4 ± 4.6 min, *p* < 0.001). Self-efficacy (87.2 ± 7.5 vs. 78.3 ± 8.4, *p* < 0.001) and course satisfaction (91.0 ± 6.8 vs. 80.5 ± 7.6, *p* < 0.001) were also higher in the AI group.

**Conclusions:**

An AI-based TEE simulation training system was associated with improved residents' simulation-based TEE learning outcomes, including image-interpretation performance, procedural assessment scores, efficiency, and perceived self-efficacy, compared with traditional teaching. However, further studies are needed to determine whether these gains translate into clinical performance in perioperative practice.

## 1 Introduction

Transesophageal echocardiography (TEE) has become a “visualized weapon” for anesthesiologists in the perioperative period. It provides real-time assessment of cardiac structure and function, hemodynamic status and intraoperative complications, thereby guiding decision-making in high-risk surgery and complex cardiac conditions (1, 2). Consequently, structured TEE training is an essential component of anesthesiology residency programs (3).

Traditional TEE teaching in residency is mainly based on didactic lectures, static image review and limited bedside observation. This approach has several limitations. First, theory and practice are often disconnected. Teaching typically follows a “theory first, cases later” pattern, with few opportunities to simulate dynamic intraoperative scenarios such as rapid diagnosis of the cause of hypotension, so residents may struggle to translate theoretical knowledge into real-time clinical decision-making (4). Second, instruction is not sufficiently individualized. Teaching commonly relies on “group lectures plus uniform assessment,” which does not target each resident's specific weaknesses in anatomical understanding or probe manipulation, and some trainees therefore progress with an incomplete foundation (5). Third, risk awareness and crisis management are difficult to cultivate. Hands-on practice on real patients cannot be repeatedly used to train management of TEE-related complications, such as esophageal injury or arrhythmia, and there is limited scope for deliberate practice in rare but critical crises (6, 7).

AI-based TEE simulation systems may help overcome these shortcomings by providing risk-free repetitive practice, allowing residents to perform probe insertion, manipulation and image acquisition without exposing patients to the risks of real procedures. Through continuous capture of operation data such as probe angle and image quality, AI algorithms can identify errors and offer targeted training tasks to strengthen weak skills, thereby enabling precision and personalization of teaching. In addition, the combination of three-dimensional anatomical visualization, synchronized ultrasound planes and AI-assisted image-interpretation modules creates an immersive and efficient learning environment that helps residents build intuitive links between probe movement, echocardiographic images and underlying pathology, shortening the learning curve (8, 9).

Although AI-assisted simulation has shown promising results in other medical disciplines, evidence for its role in perioperative TEE training remains limited. This study therefore aimed to evaluate the effectiveness of an AI-based TEE simulation training system in anesthesiology residents at Sichuan Academy of Medical Sciences & Sichuan Provincial People's Hospital. We hypothesized that, compared with traditional teaching, the AI-assisted approach would improve TEE theoretical knowledge, technical performance, efficiency and learner satisfaction.

## 2 Methods

### 2.1 Study design and participants

This single-center, prospective, randomized controlled trial was conducted in the Department of Anesthesiology, Sichuan Academy of Medical Sciences & Sichuan Provincial People's Hospital, from December 2024 to December 2025. All participant recruitment, teaching interventions, and outcome assessments were performed at this institution. The study protocol was approved by the Ethics Committee of Sichuan Academy of Medical Sciences & Sichuan Provincial People's Hospital (No: 2025-842), and written informed consent was obtained from all participants before enrollment. Co-authors from other institutions contributed to study design, data interpretation, and manuscript preparation, but were not involved in separate off-site participant enrollment or independent data collection.

Eligible participants were anesthesiology residents enrolled in the standardized residency training program who had not previously received systematic TEE training. Inclusion criteria were: (1) age 20–30 years; (2) in Postgraduate Year (PGY) 1–3; (3) willingness to participate and provide informed consent. Exclusion criteria were: (1) prior completion of a dedicated TEE course; (2) substantial prior experience with intraoperative TEE; (3) refusal to participate.

**Figure 1:**
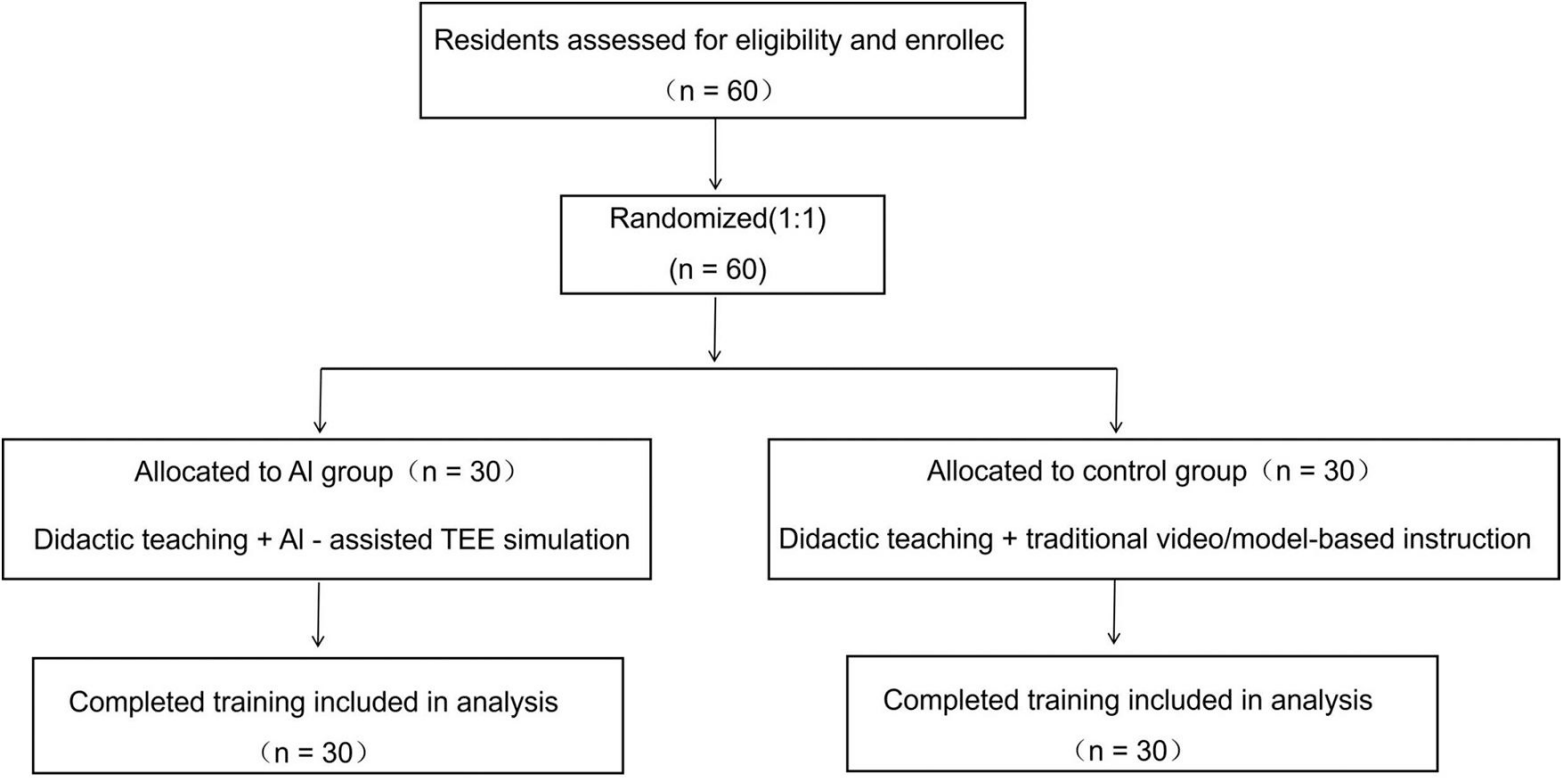
Study flow diagram. Flowchart showing resident enrollment, randomization, group allocation, completion of training, and inclusion in the final analysis.

A total of 60 residents met the criteria and were recruited. Using a computer-generated random number table, residents were assigned in a 1:1 ratio to the AI group (*n* = 30) or control group (*n* = 30) (Figure 1). Baseline characteristics included age, sex, PGY year, and educational level (bachelor vs. master's degree). As shown in Table 1, there were no significant differences between groups.

**Table 1:** Baseline characteristics of anesthesiology residents.

Variable	AI group (*n* = 30)	Control group (*n* = 30)	*p* value
Age, years	25.4 ± 1.9	25.1 ± 2.0	0.58
Male, *n* (%)	16 (53.3)	17 (56.7)	0.80
PGY-1/PGY-2/PGY-3, *n*	11/10/9	10/11/9	0.93
Bachelor/Master's degree, *n*	18/12	17/13	0.79

Data are expressed as mean ± SD or *n* (%).

All participants were receiving structured TEE training for the first time, and residents with prior formal or systematic TEE training were excluded from enrollment. Before enrollment, all trainees had completed departmental preparatory training and assessment. Baseline theoretical knowledge relevant to TEE was evaluated during this process, and no statistically significant difference was found between the two groups. Therefore, the groups were considered comparable with respect to baseline theoretical knowledge at study entry.

### 2.2 AI-based TEE simulation system

The AI-based TEE simulation system used in this study was a commercial platform developed by Qingdao Pangu Robot Co., Ltd. and designed for TEE training. Because the platform was commercially proprietary, its full technical architecture, including software version, model structure, and detailed underlying algorithmic implementation, was not completely accessible to the investigators. To improve transparency and reproducibility, we therefore describe the system in this study according to the functional components available to users and instructors during training. To improve transparency and reproducibility, we therefore describe here the functional components available to users and instructors during the study.

The platform combined conventional simulation functions, including three-dimensional (3D) anatomical visualization, probe-motion tracking, and predefined standard-view guidance, with algorithm-supported modules for performance analysis and real-time feedback. Specifically, the system provided automated assessment of image quality, recognition of target anatomical structures in acquired views, and individualized feedback generated according to each trainee's prior errors, task completion history, and performance patterns. These AI-assisted functions were integrated into a structured simulation environment that also included rule-based instructional prompts and scripted learning tasks.

The system used in this study consisted of three core components. First, a 3D anatomy visualization module with plane linkage provided a high-fidelity three-dimensional heart model displaying cardiac chambers, valves, great vessels, and surrounding structures. The position and rotation of the probe within the esophagus or stomach were shown in real time and dynamically linked to the corresponding TEE views, such as the mid-esophageal four-chamber view or the transgastric short-axis view of the left ventricle. Second, an interactive image-interpretation module generated tasks requiring identification of anatomical structures, interpretation of measurement parameters, and recognition of typical pathologies, including valvular regurgitation and regional wall-motion abnormalities. Third, a virtual hands-on simulation module incorporated a physical TEE probe mock-up connected to the virtual environment, allowing residents to practice insertion, depth adjustment, angle rotation, anteflexion, retroflexion, and acquisition of standard views. When image quality or view position was suboptimal, the system issued immediate feedback and corrective prompts. All operation logs, including probe angle, depth, time required to obtain each view, and image-quality scores, were stored in the system and could be reviewed by both the resident and the instructor.

## 3 Teaching protocol

All teaching was delivered by the same TEE-certified anesthesiologist to avoid instructor bias. The total course duration was four weeks for both groups and included didactic and practical components.

### 3.1 Didactic teaching (both groups)

Both groups received identical classroom teaching. The didactic component consisted of four 45-minute sessions covering basic TEE principles, indications and contraindications, relevant cardiac anatomy, standard views as defined by the American Society of Echocardiography and the Chinese expert consensus, as well as probe manipulation and basic measurements. One week before the first practical session, residents were provided with lecture notes, teaching images and videos to facilitate self-directed pre-class study.

### 3.2 Practical teaching—control group

The control group received the same didactic teaching as the AI group but did not have access to the AI-based simulation platform. Their practical teaching followed a traditional format consisting of instructor-led demonstrations, static teaching images, heart models, and pre-recorded operative videos. The instructor provided step-by-step explanations of probe handling, standard-view acquisition, identification of key anatomical structures, and interpretation of common hemodynamic findings. Group discussion and question-and-answer sessions were used to reinforce learning.

Residents in the control group did not use an AI-assisted simulator, did not receive automated image-quality assessment or adaptive feedback, and did not perform virtual probe-manipulation training in an interactive simulation environment. Due to operating room constraints, they also did not perform TEE on actual patients during the study period. Accordingly, the contrast in this study was between AI-assisted simulation plus didactic teaching in the intervention group and traditional didactic teaching plus passive practical instruction based on videos and heart-model demonstration in the control group.

### 3.3 Practical teaching—AI group

The AI group participated in the same didactic sessions as the control group, but their practical teaching was based on the AI-driven TEE simulation system. Practical training started with a guided orientation to the simulator interface and three-dimensional cardiac anatomy, followed by systematic practice of standard views using virtual probe manipulation. Residents then engaged in interactive image-interpretation modules focusing on structure recognition, quantitative measurements and common perioperative pathologies. Scenario-based training was also incorporated, using classic cases such as intraoperative hypotension after MitraClip implantation with coronary air embolism, which was diagnosed and managed under TEE guidance. Each resident in the AI group completed at least eight supervised simulation sessions of 45 min each over the four-week period and was encouraged to practice independently outside scheduled teaching hours.

## 4 Outcome measures

### 4.1 Standardized image-interpretation test

A written test based on the Chinese Expert Consensus on Perioperative TEE Monitoring (2020) was used to assess theoretical knowledge and image-interpretation ability. The test consisted of 50 items, including single-best-answer and image-based questions, covering identification of cardiac structures, interpretation of measurements and recognition of pathological findings. Each item was worth 2 points, yielding a total score ranging from 0 to 100. All residents completed the test immediately upon completion of the four-week course.

### 4.2 OSATS-TEE score

Two experienced TEE instructors independently scored residents' performance using the same predefined OSATS-TEE criteria. The assessors were independent of the study intervention and were not involved in participant randomization, group allocation, or training delivery. To reduce scoring bias, all assessments followed a standardized evaluation framework and were reviewed according to prespecified scoring items.

However, because the assessment modality differed between groups, complete blinding of assessors to training condition was not feasible. Residents in the AI group were evaluated in a simulator-based setting, whereas residents in the control group were evaluated using standardized video-based tasks. As a result, assessors could potentially infer the assessment context. Any discrepancies between the two assessors were resolved through discussion.

### 4.3 Key-view acquisition rate and procedure time

Residents were required to obtain 20 standard TEE views as defined in the Chinese expert consensus. For each resident, the key-view acquisition rate was calculated as the number of correctly obtained standard views divided by 20 and expressed as a percentage. Procedure time was defined as the interval from the beginning of probe manipulation to successful acquisition of all 20 views and was recorded in minutes.

### 4.4 Self-efficacy and course satisfaction

After the course, residents completed anonymous questionnaires to evaluate self-efficacy and course satisfaction. The self-efficacy questionnaire was a study-specific 10-item instrument developed according to the predefined learning objectives of the TEE curriculum. It assessed residents' perceived confidence in key domains of TEE learning, including standard-view acquisition within a limited time, image optimization, recognition of major cardiac structures, troubleshooting of difficult views, basic Doppler-related operations, identification of common pathological findings, self-correction during operation, communication of key findings, transfer of skills to new clinical scenarios, and confidence in passing the course assessment. Each item was scored on a 4-point Likert scale (1 = strongly disagree to 4 = strongly agree). The total raw score therefore ranged from 10 to 40 and was linearly transformed to a 0–100 scale for analysis, with higher scores indicating greater perceived self-efficacy.

The course satisfaction questionnaire was a study-specific 5-item instrument used to evaluate residents' perceptions of the clarity of course objectives, helpfulness of the AI-assisted feedback, appropriateness of training intensity and difficulty, adequacy of teaching support, and overall willingness to recommend the course. Each item was scored on a 5-point Likert scale, and the total raw score ranged from 5 to 25. For consistency of presentation across outcomes, this raw score was also converted to a 0–100 scale for analysis, with higher scores indicating greater course satisfaction.

Both questionnaires were developed specifically for this study according to the predefined educational objectives of the training program. They were used as supplementary learner-reported outcome measures rather than previously established standardized scales.

### 4.5 Statistical analysis

All data were analyzed using SPSS 22.0 software (version 22.0, IBM Corp., Armonk, NY, USA). Continuous variables were presented as mean ± standard deviation, and categorical variables were expressed as counts and percentages. Baseline characteristics between the two groups were compared using independent-samples t tests for continuous variables and chi-square tests for categorical variables. Post-training outcomes, including test scores, OSATS-TEE scores, key-view acquisition rate, procedure time, self-efficacy, and course satisfaction, were compared between groups using independent-samples *t* tests. A two-sided *p* value < 0.05 was considered statistically significant. Sample size was estimated for the primary outcome (standardized image-interpretation test score). Assuming an effect size of 0.80, a two-sided *α* of 0.05, and 80% power, at least 26 residents were required per group. To allow for possible attrition, 30 residents were enrolled in each group (total *n* = 60). The standardized image-interpretation test was prespecified as the primary outcome, whereas the remaining outcomes were considered secondary exploratory outcomes. No formal adjustment for multiple comparisons was applied; therefore, results for secondary outcomes should be interpreted cautiously because of the increased risk of type I error. Because no formal pre-course assessment was performed, baseline-adjusted or change-score analyses were not possible, and the study was analyzed as a randomized post-test comparison. In addition to *p* values, the between-group difference for the primary outcome was summarized using the mean difference, 95% confidence interval (CI), and Cohen's d effect size.

## 5 Results

### 5.1 Baseline characteristics

Sixty residents were enrolled and completed the study (AI group, *n* = 30; control group, *n* = 30). As shown in Table 1, there were no significant between-group differences in age, sex, PGY year, or educational level (all *p* > 0.05).

### 5.2 Standardized image-interpretation test

**Figure 2:**
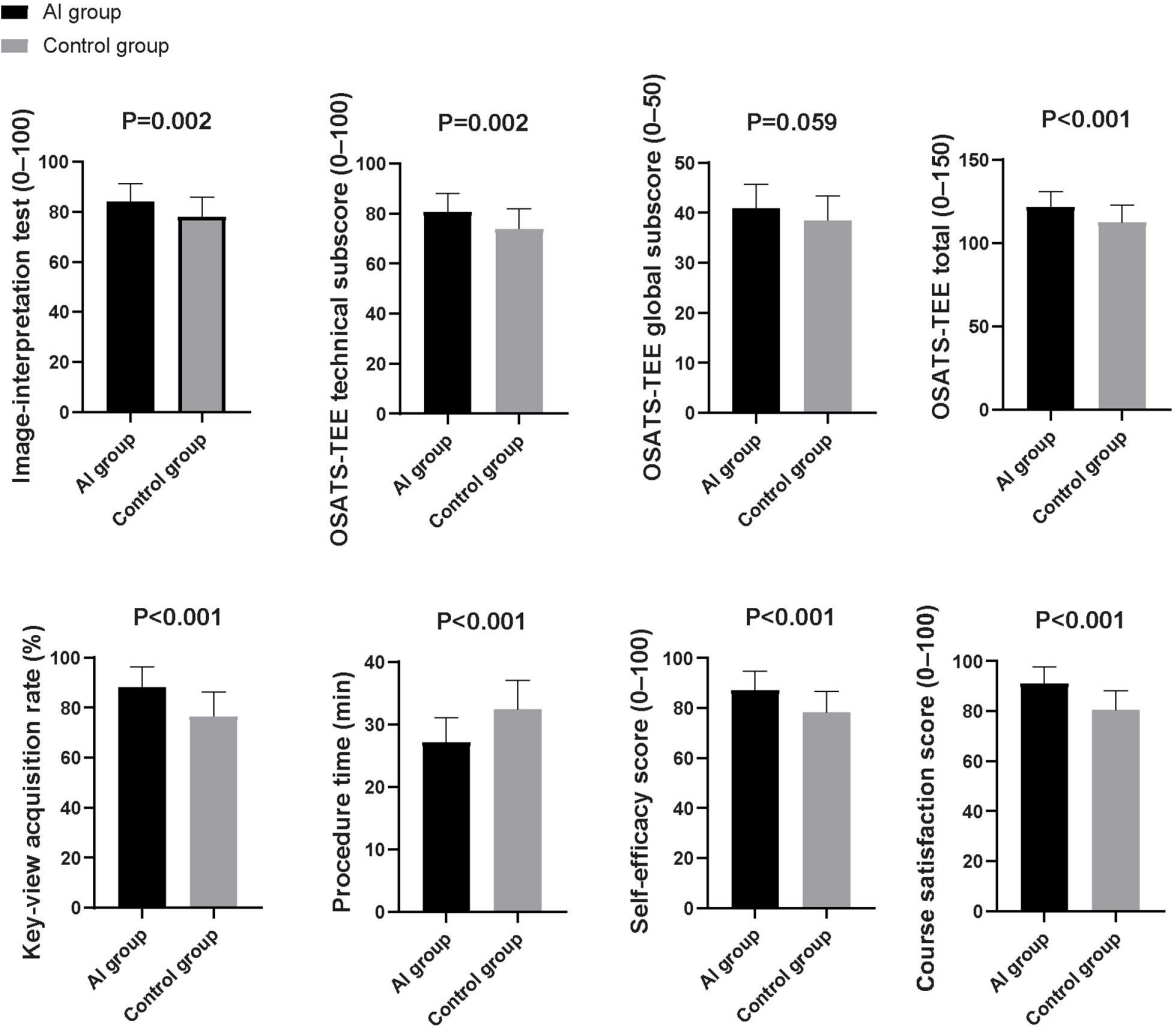
Comparison of post-training outcomes between the AI group and control group. Bar charts showing post-training performance in the AI group and control group, including image-interpretation test score, OSATS-TEE technical subscore, OSATS-TEE global subscore, OSATS-TEE total score, key-view acquisition rate, procedure time, self-efficacy score, and course satisfaction score. Data are presented as mean ± SD. *P* values indicate between-group comparisons.

After the 4-week course, standardized image-interpretation test scores were higher in the AI group than in the control group (84.3 ± 6.9 vs. 78.1 ± 7.8, mean difference 6.2 points, 95% CI: 2.4–10.0, *t* = 3.12, *p* = 0.002; Cohen's *d* = 0.84; Table 2, Figure 2).

**Table 2:** Comparison of image-interpretation and OSATS-TEE scores after training.

Outcome	AI group (*n* = 30)	Control group (*n* = 30)	*t* value	*p* value
Image-interpretation test (0–100)	84.3 ± 6.9	78.1 ± 7.8	3.12	0.002
OSATS-TEE technical subscore (0–100)	80.6 ± 7.5	73.9 ± 8.0	3.28	0.002
OSATS-TEE global subscore (0–50)	40.9 ± 4.8	38.5 ± 4.9	1.93	0.059
OSATS-TEE total (0–150)	121.5 ± 9.2	112.4 ± 10.1	3.53	<0.001

Values are mean ± SD. Replace numbers with your real data before submission.

### 5.3 OSATS-TEE scores

OSATS-TEE total scores were also higher in the AI group (121.5 ± 9.2 vs. 112.4 ± 10.1, *t* = 3.53, *p* < 0.001; Table 2, Figure 2). However, because the OSATS-TEE assessment was performed under different formats in the two groups, these findings should be interpreted as differences in training-context-specific assessment performance rather than definitive evidence of superior technical skill. The technical subscore showed a similar pattern, whereas the difference in the global subscore did not reach statistical significance (Table 2).

### 5.4 Key-view acquisition rate and procedure time

The key-view acquisition rate was greater in the AI group (88.2% ± 8.1% vs. 76.5% ± 9.7%, *t* = 4.60, *p* < 0.001), while procedure time was shorter (27.1 ± 3.9 min vs. 32.4 ± 4.6 min, *t* = –4.73, *p* < 0.001; Table 3, Figure 2).

**Table 3:** Key-view acquisition rate, procedure time, self-efficacy and course satisfaction.

Outcome	AI group (*n* = 30)	Control group (*n* = 30)	*t* value	*p* value
Key-view acquisition rate (%)	88.2 ± 8.1	76.5 ± 9.7	4.60	<0.001
Procedure time (min)	27.1 ± 3.9	32.4 ± 4.6	−4.73	<0.001
Self-efficacy score (0–100)	87.2 ± 7.5	78.3 ± 8.4	4.23	<0.001
Course satisfaction score (0–100)	91.0 ± 6.8	80.5 ± 7.6	5.41	<0.001

### 5.5 Self-efficacy and course satisfaction

Learner-reported self-efficacy and course satisfaction were both higher in the AI group than in the control group (self-efficacy: 87.2 ± 7.5 vs. 78.3 ± 8.4, *p* < 0.001; course satisfaction: 91.0 ± 6.8 vs. 80.5 ± 7.6, *p* < 0.001; Table 3, Figure 2).

## 6 Discussion

Traditional teaching models are limited by scarce hands-on resources and fragmented teaching time. In addition, the standardized pace of instruction often fails to match the learning needs of residents with different levels of baseline knowledge, resulting in a prolonged learning cycle and low resource utilization efficiency (10, 11). In contrast, AI-based teaching models overcome the constraints of practical resources by allowing residents to repeatedly practice probe manipulation and image acquisition without limitation, while real-time feedback and adaptive training support may enable more individualized learning (12, 13).

Importantly, the potential advantage of the present intervention was not simulation alone, but the integration of algorithm-supported personalization within the simulation environment. Compared with standard simulation, which often provides the same predefined sequence of practice tasks to all learners, the AI-based system in this study offered automated image-quality assessment, recognition of target anatomical structures, and adaptive feedback based on each trainee's prior errors and completion history. This may have allowed residents to receive more individualized guidance, focus more efficiently on specific weak points, and engage in a more responsive learning process. Such personalized feedback may be particularly relevant in TEE training, where errors in probe manipulation, anatomical orientation, and view optimization often differ substantially across learners. Therefore, the observed educational benefit may reflect not only repeated simulator exposure, but also the added value of AI-assisted performance analysis and tailored feedback.

In this study, we applied an AI-based TEE simulation training system to transesophageal echocardiography education for residents and evaluated their mastery of theoretical knowledge and practical skills. The results suggest that the AI-based TEE simulation system, particularly its personalized feedback and adaptive training support, improved residents' theoretical understanding and simulation-based procedural performance. Because it does not rely on a physical TEE probe or clinical patients, residents can practice probe manipulation, image acquisition, and positional adjustments at any time, with unlimited repetitions. This avoids the medical risks and resource limitations inherent in clinical practice, enables rapid consolidation of fundamental skills, and offers significant advantages in TEE training. These findings are consistent with those of Kim TE (14, 15), who reported similar benefits of AI-based simulation systems in regional anesthesia training.

Furthermore, virtual simulation technology utilizes human-body and equipment sensing to provide residents with realistic three-dimensional anatomical images, creating an immersive learning environment that enhances focus and learning efficiency (16).

In addition, the AI-based simulation system removes the constraints of time and location in resident education, allowing skill reinforcement outside the operating room at any moment, thereby optimizing teaching resources and improving accuracy and stability during real procedures (17). Rothkrug et al. similarly demonstrated favorable educational outcomes when applying AI-based simulation systems to anesthesia crisis training scenarios (18). By recreating high-fidelity clinical environments, providing personalized training pathways, and allowing risk-free repetitive practice, the AI-based simulation system reduces clinical trial-and-error costs and enables residents to train repeatedly in a virtual setting without any medical risk (19).

However, it is important to emphasize that the present study evaluated training effectiveness within a simulation-based educational environment rather than actual clinical TEE practice. Although the AI group showed better post-training knowledge scores, technical performance, efficiency, and learner-reported confidence, these improvements cannot be assumed to directly translate into superior intraoperative performance, improved patient safety, or better clinical outcomes. Demonstrating true clinical skill transfer will require future studies that assess supervised performance on real patients and examine whether simulator-based gains are maintained and applied effectively in the operating room. In addition, the OSATS-TEE assessment was not performed under identical conditions in the two groups. Therefore, the higher OSATS-TEE scores observed in the AI group should be interpreted as higher performance on a training-context-specific assessment rather than conclusive evidence of superior technical skill. Similarly, although the image-interpretation test is educationally relevant, it remains a controlled assessment rather than a direct measure of intraoperative clinical performance. In addition, learner-reported self-efficacy and course satisfaction should be interpreted cautiously, as these were study-specific supplementary measures rather than previously validated instruments. Moreover, because multiple secondary outcomes were analyzed without formal adjustment for multiple comparisons, these findings should be interpreted as supportive and exploratory rather than definitive.

The potential generalizability of these findings should also be interpreted with caution. Although the educational advantages observed in this study may be relevant to other institutions, the magnitude of benefit may depend on local training structure, faculty experience, simulator availability, and residents' baseline exposure to perioperative echocardiography. In addition, our participants were general anesthesiology residents rather than trainees specifically focused on cardiac anesthesia or cardiac surgery. Trainees in cardiac-oriented programs may have different baseline knowledge, stronger clinical motivation for TEE learning, and more frequent exposure to relevant cases, which could influence both learning curves and the incremental value of simulation-based teaching. Furthermore, differences across healthcare systems, including training duration, access to simulation resources, and the role of TEE in routine perioperative practice, may also affect how readily this teaching model can be implemented and how effective it may be in other settings. Therefore, multicenter studies involving diverse trainee populations and institutional contexts are warranted.

### 6.1 Limitations

This study has several limitations. First, although participants had not received formal TEE training, the assumption of a “zero baseline” may have been too strong, because prior informal exposure, such as related rotations or self-directed learning, was not measured. Second, no formal pre-course assessment was performed, which limited evaluation of within-group improvement, prevented baseline-adjusted analyses, and did not allow full exclusion of pre-existing differences. Third, the OSATS-TEE assessment was conducted under non-identical conditions between groups, so these results should be interpreted as training-context-specific rather than definitive evidence of superior technical skill. Fourth, all outcomes were simulation-based, and transfer to real perioperative TEE practice was not assessed. Fifth, the simulator was a proprietary commercial system, and detailed technical information about its underlying algorithms was unavailable, which limits reproducibility. Finally, this was a single-center study with a relatively small sample size and no long-term follow-up, which may limit generalizability and assessment of skill retention.

## 6 Conclusions

The application of an AI-based TEE simulation training system in anesthesiology residency education was associated with improved simulation-based TEE learning outcomes, including image-interpretation performance, training-context-specific assessment scores, efficiency, self-efficacy, and course satisfaction, compared with traditional teaching alone. AI-driven simulation provides a safe, high-fidelity, and personalized training environment and may serve as a valuable supplement to conventional TEE teaching. However, further studies are needed to determine whether these simulation-based educational gains translate into improved clinical performance and patient care in real operating-room settings.

## Data Availability

The original contributions presented in the study are included in the article/Supplementary Material, further inquiries can be directed to the corresponding author/s.
